# Hydropneumothorax: A Presentation of Infected Bronchogenic Cyst

**DOI:** 10.7759/cureus.38569

**Published:** 2023-05-05

**Authors:** Shreya Garg, Kanishk Aggarwal, FNU Anamika, Avi Kumar

**Affiliations:** 1 Internal Medicine, Dayanand Medical College and Hospital, Ludhiana, IND; 2 Internal Medicine, University College of Medical Sciences, New Delhi, IND; 3 Respiratory Medicine, Fortis Escorts Heart Institute, New Delhi, IND

**Keywords:** bronchogenic cyst, thoractomy, mediastinal mass, congential lung lesions, hydropneumothorax

## Abstract

Bronchogenic cysts are closed sac-like cystic lesions resulting from abnormal budding of the primitive foregut during the early development of the alimentary and respiratory systems. We describe the case of a 54-year-old man who presented to the emergency department with complaints of fever, chills, shortness of breath, and a productive cough with intermittent hemoptysis for the past two to three months. Initial workup revealed a right lung hydropneumothorax with complete atelectasis of the right lung and a mass effect on the left lung. During intercostal drainage, pleural fluid analysis revealed empyema with *E. coli* treated with antibiotics. However, the symptoms persisted after five days of antibiotic treatment and drainage. A multidisciplinary team of thoracic surgeons, anesthesiologists, and pulmonologists was assembled due to the non-resolving nature of the lung abscess. The patient underwent a right middle lobe lobectomy with decortication via open thoracotomy, and a bronchogenic cyst, an uncommon cause of the lung abscess, was suggested by histopathological analysis.

## Introduction

Congenital lung lesions comprise a diverse spectrum of rare but clinically significant lesions ranging from congenital cystic adenomatoid malformations to bronchopulmonary sequestrations, congenital lobar emphysema, and bronchogenic cysts [1]. Bronchogenic cysts are closed sac-like cystic lesions resulting from abnormal budding of the primitive foregut during the early development of the alimentary and respiratory systems [2,3]. Furthermore, they are exceedingly rare, with a prevalence rate of 1 in 42000 and 1 in 68000 [4]. They are more common in males than females, appear more frequently in the third and fourth decades of life, and account for 6%-15% of all primary mediastinal masses [5]. The clinical presentation of the bronchogenic cyst is variable, from asymptomatic to fever, cough, hemoptysis, and chest pain, depending upon various complications like fistula formation, pneumonia, or lung abscess [6]. It can also be large enough to cause pressure on adjacent vital structures such as the tracheal carina, the lung, or the esophagus. It can even be life-threatening due to airway obstruction causing respiratory distress in the pediatric population [7]. The diagnosis is often made using chest radiographs and CT scans showing spherical or oval masses with smooth outlines [8]. Despite the value of various noninvasive diagnostic studies, a definitive diagnosis can only be made by surgical excision and tissue biopsy [9]. In this case report, we present a patient treated for a non-resolving empyema caused by a bronchogenic cyst congenital lung lesion that is a rare cause of lung abscess and requires effective collaboration between medical and surgical specialties.

## Case presentation

A 54-year-old man presented to the emergency department with complaints of worsening dyspnea, intermittent hemoptysis, a productive cough for two to three months, and a fever with chills for the last 10 days. He experienced progressive dyspnea, even at rest, along with intermittent, dull, non-radiating chest pain on the right side without any aggravating or alleviating variables. The patient first had a non-productive cough that later became more intense and productive, coughing up about a cup's worth of sputum on three occasions with occasional hemoptysis. He developed a fever and chills 10 days before hospitalization. He had no medical history of immunodeficiency, IV drug use, or alcohol addiction and did not have night sweats, palpitations, or weight loss. There was no significant past medical or family history.

**Figure 1:**
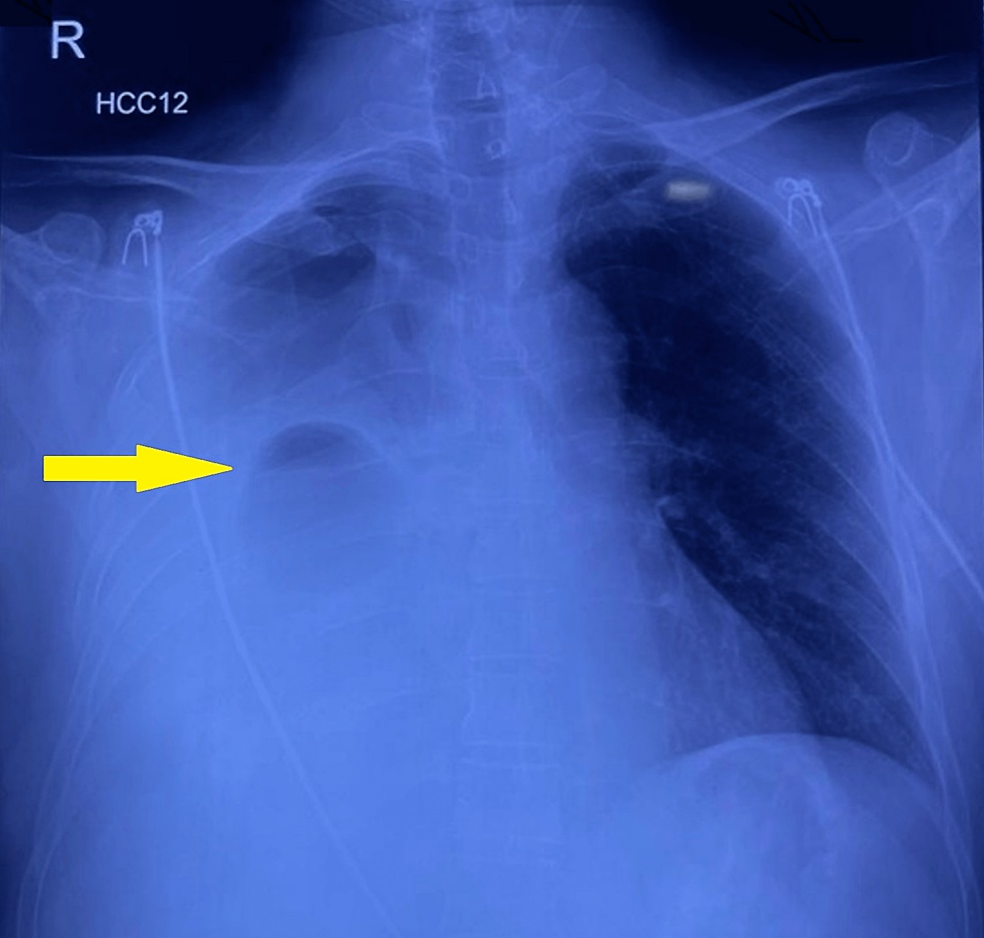
Chest X-ray showing right middle zone cavitary lesion with right-sided pleural effusion.

On examination, the patient was awake, alert, and oriented but in respiratory distress. He was afebrile on presentation, and his pulse rate was 116/minute, regular, and of good volume. His blood pressure was 112/56mmHg, his respiratory rate was 28/minute, and his oxygen saturation was 88% at room air. On chest inspection, there was asymmetry in the chest expansion, where his right hemithorax expanded less than the left. Percussion revealed a dull note over the right middle and lower zones, anteriorly and posteriorly. On auscultation, the breath sounds were significantly diminished on the right side. There were no significant examination findings pertaining to other systems. The patient underwent a chest X-ray (Figure 1), which revealed a right-sided cavitary lesion in the right middle zone, an opaque right phrenic angle, and diffuse opacification of the right hemithorax. There was a slight mass effect towards the left lung; however, the left lung was normal.

**Figure 2:**
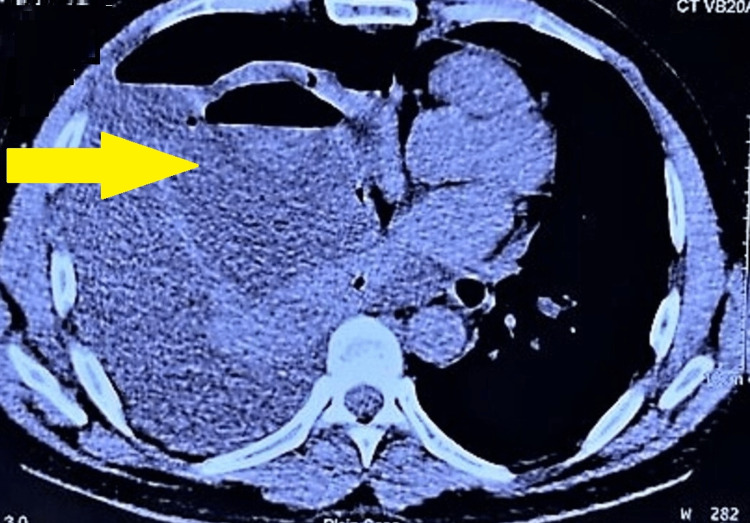
HRCT showing right-sided hydropneumothorax and significant right-sided pleural effusion suggestive of empyema.

His arterial blood gas findings: pH= 7.46, pCO2 =29.8, pO2= 49.7 implied respiratory alkalosis secondary to tachypnoea. Later, a high-resolution chest CT scan (Figure 2) was suggestive of a right middle and lower lobe cystic lesion with an air-fluid level, a leftward cardio-mediastinal shift and mass effect on the lower trachea and left lung, and complete atelectasis of the right lung, making lung abscess and hydatid cyst two of our main differentials.

**Figure 3:**
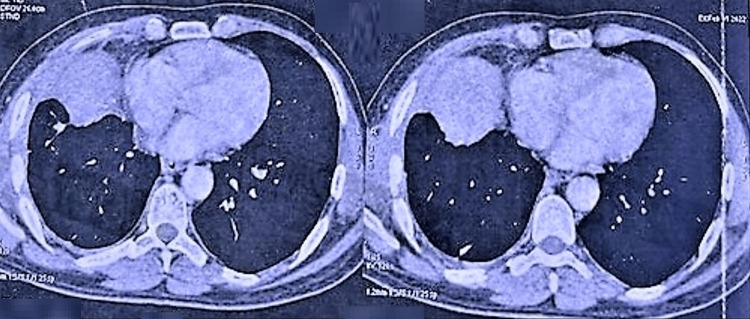
HRCT: Post-right middle lobe lobectomy and decortication via open thoracotomy

Immediately, the patient was started on broad-spectrum antibiotics for the treatment of lung abscess, and right lung intercostal chest tube insertion was done. Thick purulent fluid was drained with adenosine deaminase of 94 IU/L (cutoff 100; >100= TB effusion) and a high LDH of 14804 units (LDH>1000- indicative of infection), indicating empyema. Pleural fluid culture and sensitivity were positive for *Escherichia coli* and were sensitive to fosfomycin and tigecycline. Antibiotics were modified accordingly. Considering the chronic presentation, cavitary pulmonary tuberculosis was a substantial differential. However, the gene experts for Mycobacterium tuberculosis and rifampicin resistance came out negative. Hydatid serology also came out negative. The patient was treated with appropriate antibiotics and intercostal drainage but showed no signs of clinical recovery. So, a step towards surgical excision was considered, and multispeciality treatment with thoracic surgeons was taken on board. The patient underwent a right middle lobectomy with decortication via open thoracotomy, and the procedure went uneventfully. Figure 3 shows the post-surgical CT findings.

The histopathology report showed cystic tissue lined by ciliated pseudostratified columnar cells with sero-mucinous glands, hyaline cartilage, and muscle tissue with acute and chronic inflammatory infiltrates. There were no granulomas or evidence of malignancy, and the report suggested a bronchogenic cyst, a rare cause of secondary lung abscess. The patient was discharged after clinical improvement post-procedure and reported resolving symptoms on follow-up. 

## Discussion

Bronchogenic cysts present as a congenital anomaly that usually arises during the sixth week of gestation from abnormal or late budding of the ventral lung bud or the tracheobronchial tree during development. It is lined by the respiratory epithelium, composed of cartilage, smooth muscle, fibrous tissue, and mucous glands [4]. It is the most common primary cyst of the mediastinum located in the thorax, either in the mediastinum or in the pulmonary parenchyma. As a result of aberrant mesenchymal growth during embryogenesis, they can occasionally be seen subcutaneously as cutaneous lesions or even within the diaphragm [10]. Rare cases of intraspinal bronchogenic cysts have also been reported, leading to symptoms of compressive myelopathy [11].

The clinical presentation of the bronchogenic cyst is variable based on age, cyst location, and probably size [12]. Symptoms can range from stridor to esophageal symptoms (particularly feeding problems), critical airway obstruction, and severe lower respiratory symptoms (cough, fever, dyspnea, and hypoxia) in the pediatric population [6,13]. It can also present as fever, cough, hemoptysis, and chest pain in adults, which often indicate the development of complications [14]. Approximately 45% of patients with bronchogenic cysts develop severe fatal complications like life-threatening hemoptysis, pneumothorax, pleuritis, esophageal compression, and post-obstructive pneumonia [6]. Some rare complications include superior vena cava syndrome and fatal myocardial infarction secondary to compression of the left main coronary artery [15,16]. 

An array of pathologies can mimic bronchogenic cysts. The differential diagnosis of bronchogenic cysts includes neoplasms, granulomas, hematomas, vascular malformations, lung sequestration, lung abscesses, infected bullae, hydatid cysts, inflammatory lymphadenopathy, and neuro-enteric, pericardial or esophageal duplication cysts [16]. Given the unusual and insignificant character of the presentation as well as the potential for life-threatening consequences with wide pathologies in differentials, prompt imaging and surgical excision of all suspected bronchogenic cysts are essential. 

Chest radiographs and CT scans are the most valuable diagnostic studies [17]. A chest radiograph usually reveals a smooth, round, well-demarcated, non-calcified mass in the mediastinum or lung [17]. Chest radiographs often become limited when the size is too small or in cases of infection or rupture of a cyst with the development of an air-fluid level or variations in the density of the cyst caused by calcification or pneumonia. Usually, most bronchogenic cysts are homogeneous masses and have a low CT number of 0-20 Housefield units, but cysts with high density because of increased calcium content, blood, or greater protein content of the fluid often result in high CT numbers and may appear solid, leading to a diagnostic dilemma. MRI is an advantageous technique for providing supplementary diagnostic data. In a typical noninfected cyst containing serous fluid, T1-weighted images show a low signal and T2-weighted images show a high signal, whereas cysts with increased protein content, hemorrhage, or mucosal components tend to show moderate to high T1-weighted signals on MRI. In cases where CT suggests a solid tumor, MRI becomes an essential diagnostic tool to make an almost precise preoperative diagnosis and differentiate between solid tumors and cysts by T1-weighted image signal showing moderate to high strength and T2-weighted image showing high signal strength [17,18]. Despite the value of various noninvasive diagnostic studies, a definitive diagnosis can only be made by surgical excision and tissue biopsy [9]. 

Early surgical intervention is recommended for treatment and carries an excellent prognosis [19,20]. Most adult patients will develop symptoms and complications if left untreated, and even malignant changes can be missed. Surgical excision is usually via a posterolateral thoracotomy or median sternotomy with intrapulmonary cysts requiring segmental or lobar resection. Another suggested alternative to surgical excision via thoracotomy is video-assisted thoracoscopy resection [17]. The acceptance of transbronchial, transesophageal, transtracheal, extrapleural, and percutaneous aspiration as alternatives to operation is low [16].

## Conclusions

Most bronchogenic cysts are asymptomatic and discovered incidentally in routine imaging studies. They become symptomatic when the cyst gets infected, any other complication arises, or it becomes large enough to cause compression on nearby structures. As there is high variability in their location, the signs and symptoms of bronchogenic cysts are highly variable. Various diagnostic imaging modalities, including chest X-rays and CT scans, are available. Here we present a rare case of the bronchogenic cyst as a lung abscess that was surgically treated by open thoracotomy and lobectomy. We recommend that symptomatic and asymptomatic patients be surgically treated, either through thoracotomy or thoracoscopy, as there are high chances of complications and the development of malignant features in cases of asymptomatic bronchogenic cysts.
